# Wheat Landraces Are Better Qualified as Potential Gene Pools at Ultraspaced rather than Densely Grown Conditions

**DOI:** 10.1155/2014/957472

**Published:** 2014-05-11

**Authors:** Elissavet G. Ninou, Ioannis G. Mylonas, Athanasios Tsivelikas, Parthenopi Ralli, Christos Dordas, Ioannis S. Tokatlidis

**Affiliations:** ^1^School of Agriculture, Aristotle University of Thessaloniki, 541 24 Thessaloniki, Greece; ^2^International Centre for Agricultural Research in the Dry Areas, Genetic Resources Section, BP 435, Menzah 1, 1004 Tunis, Tunisia; ^3^Hellenic Agricultural Organization—DEMETER, Agricultural Research Center of Northern Greece, Thermi, 570 01 Thessaloniki, Greece; ^4^Department of Agricultural Development, Democritus University of Thrace, 68200 Orestiada, Greece

## Abstract

The negative relationship between the yield potential of a genotype and its competitive ability may constitute an obstacle to recognize outstanding genotypes within heterogeneous populations. This issue was investigated by growing six heterogeneous wheat landraces along with a pure-line commercial cultivar under both dense and widely spaced conditions. The performance of two landraces showed a perfect match to the above relationship. Although they lagged behind the cultivar by 64 and 38% at the dense stand, the reverse was true with spaced plants where they succeeded in out-yielding the cultivar by 58 and 73%, respectively. It was concluded that dense stand might undervalue a landrace as potential gene pool in order to apply single-plant selection targeting pure-line cultivars, attributable to inability of plants representing high yielding genotypes to exhibit their capacity due to competitive disadvantage. On the other side, the yield expression of individuals is optimized when density is low enough to preclude interplant competition. Therefore, the latter condition appears ideal to identify the most promising landrace for breeding and subsequently recognize the individuals representing the most outstanding genotypes.

## 1. Introduction

The growing global population set new challenges to agricultural production that should meet higher food demands by less arable land and under variable patterns of rainfall that jeopardize the successful cultivation of annual plants [1]. In other words, increasing pressure is placed on agricultural systems to supply more food under unpropitious circumstances. Future climate change scenarios suggest that abiotic stress may occur at unexpected stages of plant development, thus decreasing yield consistency [2]. Global warming scenarios could reduce wheat productivity in zones where optimal temperature already exists, potentially increasing food insecurity and poverty [3]. As a consequence, breeding of new cultivars ought to play a crucial role in the days ahead in order to combat these challenges. However, in predominantly self-pollinated species like wheat, no long-term investments are attracted when farmers use their own seeds [1]. Hence, the utilization of the genetic variability of traditionally cultivated and locally adapted farmer varieties, the so-called landraces, may offer an alternative short-term channel. Such germplasms constitute valuable gene pools [4] and presumably consist of mixtures of fairly homogeneous plants offering thus the chance to immediately isolate single-plant progenies targeting pure-line cultivars.

Recently, a lot of effort has put into collecting, organizing, studying, and analyzing wheat landraces, whose potential for improved deployment and exploitation and incorporation of their positive qualities into new cultivars was explored [4]. The identification of the most promising landraces to employ breeding and build pure-line cultivars is the first crucial step in accomplishing a successful progress through selection. Since, a negative relationship between yielding and competitive ability has been reported [5–8], the aim of this study was to assess whether the normally used densities, that enforce intragenotype competition or the absence of competition, offer more chances to recognize the promising landraces to employ breeding.

## 2. Materials and Methods

The study pertains to six wheat landraces, plus one cultivar (cv. Simeto) as check (see Table 1). The cultivation of landraces “Nteves” and “Grinias” is reported at the beginning of 20th century [9]. These six landraces had been important varieties for wheat production in Greece till the 80s, when acquired during the large wheat collection of the Greek Gene Bank, just before they have been replaced by modern wheat cultivars (P. Ralli, personal communication). They were tested in a three-year field experimentation as winter-type wheat (sowing time, middle of November), at the farm of the Agricultural Research Centre of Northern Greece, Hellenic Agricultural Organization-DEMETER, Thessaloniki (2008/09 and 2009/10), and at the farm of Aristotle University of Thessaloniki (2011/12). The landraces were initially evaluated at the typical farming density (TFD), that is, 400–500 plants m^−2^, and under low-input regime, without fertilizers and herbicides, according to the cultivation techniques that growers are following in the area of the landrace origination. The previous crop in the field was vetch and the weed control was obtained by manual hoeing. Additionally, an evaluation under a conventional regime at the TFD was conducted, where weed control was accomplished through application of preemergent herbicide (Tribenuron methyl and diclofop methyl) and hand weeding. The basic fertilization applied at sowing was 48 kg ha^−1^ N and 60 kg ha^−1^ P_2_O_5_ in the form of phosphate ammonium (16-20-0). Additionally, 100.5 kg ha^−1^ N was applied in the middle of March as nitrate ammonium (33.5-0-0). Lastly, the experimentation was carried out at an ultralow density (ULD), where the fertilization and weed control were implemented according to the above conventional.

**Figure 1:**
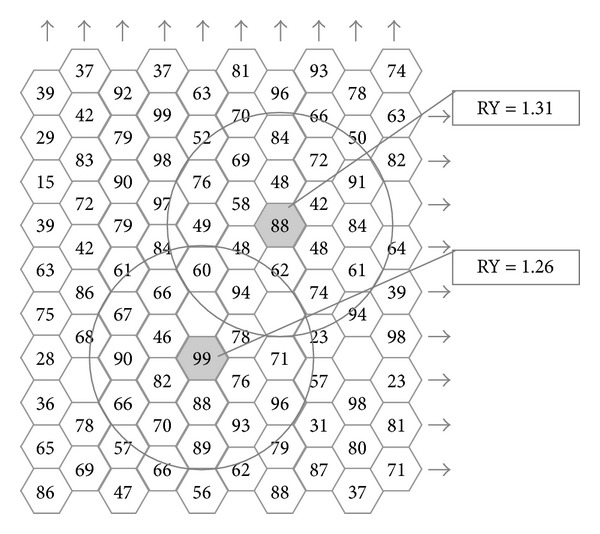
Single-plant qualification within a population allocated according to the honeycomb pattern [10]. Value of each plant is measured through its relative yield ($x/{\overset{-}{x}}_{r}$); that is, absolute yield (*x*) of the central plant is divided by the mean yield (${\overset{-}{x}}_{r}$) of all the plants included in the circle of a chosen size (up to 19 plants in this hypothetical example, missing plants do not exert any effect on the condition they are positioned widely enough to exclude interplant competition). The upper central plant is granted on relative yield better than the lower central plant (1.31 versus 1.26), even though worse on absolute yield (88 versus 99 g).

During the growing seasons of 2008/09 (low-input trial) and 2009/10 (conventional system), the six landraces and their check were sowed at a rate of 160 kg ha^−1^ targeting the TFD of 400-500 plants m^−2^, along with additional 29 landraces from the same collection and 14 commercial cultivars. The experimental design was the one-factor randomized complete block (RCB), with three replications per entry. Each plot consisted of seven rows of 2 m long and 25 cm interrow distance, with the five central rows finally harvested (2.5 m^2^). Analysis of variance was conducted for yield per area and over the two regimes. During the 2011/12 season a replicated-7 (R-7) honeycomb experiment was established including 70 plants per entry, with interplant distance of 100 cm (ULD of 1.15 plants m^−2^). Such a density was assumed to preclude any plant-to-plant interference for resources (absence of competition). Each hill was over-planted and thinned to one seedling in the middle of February, and individual plants were harvested separately. The mean yields per plant were compared by the* t*-test for independent samples and different standard deviations. Aiming to evaluate the landraces as potential starting material for the development of new pure-line cultivars, single plant selection was simulated at the ULD. The procedure qualifies each plant for relative yield, that is, the ratio of its absolute yield over the mean yield of the plants involved within a circle of chosen size the centre of which is occupied by the plant in question [10]. A hypothetical example is presented in Figure 1 using a circle size of 19 plants (18 plants surrounding the one under consideration).

## 3. Results

The analysis of variance for the two densely grown experiments revealed significant *F* values (*P* < 0.001) for genotype grain yield. The interaction of genotype by input regime was significant, depicting different genotype response and rank in the two trials. The 50 genotypes averaged grain yield of 773–4,162 kg ha^−1^ at the low-input field and 740–4,467 kg ha^−1^ at the conventionally grown field. On average the landraces lagged behind the cultivars by 42 and 51%, respectively. On the over season performance, the best landrace named Atsiki-4 (Table 1) yielded slightly less than the worst commercial cultivar, that is, 2,219 versus 2,345 kg ha^−1^.

**Figure 2:**
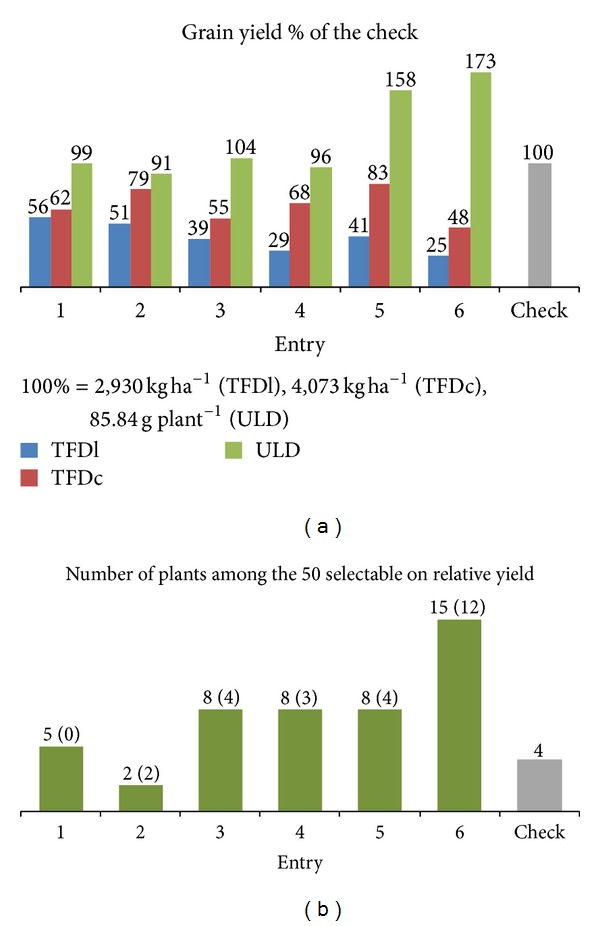
(a) Yield performance of the six landraces (1–6) and the check cultivar at the typical farming density under the low-input (TFDl) and conventional regime (TFDc), as well as at the ultralow density (ULD). (b) The number of selectable plants of each genotype on the relative yield at the ULD (in parenthesis the number of plants exceeding the highest check relative yield).

As far as the six landraces that pertain to both density regimes are concerned, Figure 2(a) illustrates the grain yield performance at the TFD comparatively with that at the ULD. At the TFD and over the input regime, all the six landraces lagged behind the check significantly by 36 up to 64%. However, at the ULD a different calculation was drawn. Four out of the six landraces managed to reach the 91–104% of the check and no one was significantly inferior. The landrace 5 and the poorest performing at the TFD landrace 6 succeeded in out-yielding significantly (*P* < 0.001) the check cultivar by 58 and 73%, respectively.

Assuming that the 50 plants were selectable on the basis of their relative yield on a circle size of 61, four of them belonged to the check while each landrace gave more outstanding plants, excepted for the landrace 2 (Figure 2(b)). The landrace 6 gave 15 selectable plants, followed by the landraces 3, 4, and 5 each involving 8 selectable plants. Assuming the highest check relative yield as the baseline (i.e., 1.52), half of the selectable plants exceeded this value (1.53–2.44).

## 4. Discussion

The results at the TFD could be interpreted as no landrace merits further consideration as breeding source. However, it was assumed as possible misinterpretation whether the negative relationship between genotype yield potential and competitive ability was present [5–8]. The hypothesis of negative association led to the decision that the next step should be an investigation under conditions that eliminate any essential influence of the interplant competition, that is, at the ULD. For this purpose, six landraces originating from differing Greek areas (Table 1) were chosen, that varied in yield at the TFD including the best and the worst ones, plus one of the most popular cultivars used for conventional cultivation. The reverse relevant to the check status of the two landraces coded 5 and 6 (Figure 2(a)) could be explained, if the speculation of the negative relationship between yield and competitive ability is taken into account. In essence, the ecosystems created in this study mirrored the three ones defined by Donald and Hamblin [11]. At the ULD the “isolation environment” regardless of the germplasm, whereas at the TFD the “competition environment” in case of the landraces and the “crop environment” in case of the check cultivar. At the isolation environment the widely spaced plants do not interfere for resources, and grow absolutely on individual potential and the available inputs. Therefore, competitive ability of individuals is of no importance and the condition optimizes the phenotypic expression of each genotype [10]. The competition environment comprises crowded plants of various genotypes. Within a mixture of genetically dissimilar individuals a part of genotypes may represent strong competitors but low yielders (Cy) at one extreme and weak competitors but high-yielders (cY) at the other [12]. In sequence of the prevailing intergenotype competition, plants share unequally the limited resources with Cy to have an advantage over the neighbouring cY. Accounting for genetic competition by the acquired competition environmentally induced (i.e., uneven germination, soil heterogeneity, pathogens, and insects), the advantage of Cy over cY might strengthen [10]. In other words, large individuals are able to obtain more resources than their share and to suppress the growth of smaller individuals. Consequently, intense crowding severely restrains yielding capacity of cY individuals, resulting thus in poor overall performance. At the ULD instead, the cY plants fully express their high yield potential and boost the overall landrace performance. The hypothesis justifies the poor average landrace performance at the TFD and perfectly applies for the two landraces coded 5 and 6 that performed the worst at the TFD and the best at the ULD. Lastly, dense stand of the check cultivar reflects intragenotype competition with individual plants striving “equally” for the limited resources, the so-called crop environment. At the crop environment, the determinant factors are the ability of the genotype to withstand in obtaining acquired variance plus the crop management to abate the environmentally induced differences. Whether these two prerequisites are met, cY genotypes of advanced genetic background and buffering ensure top yield per area in pure stand [10]. The review of 362 wheat field studies revealed a significant positive relationship between stand uniformity and mean yield [13], implying that the stand uniformity in field crops is an important mechanism for increasing grain yield [7]. Therefore, the superior yield performance of cv. Simeto at the TFD is attributable to its genetic homogeneity.

The hypothesis of inverse connection between yield and competitive ability is also justified by previous studies. A vetch (*Vicia sativa*) landrace was tested along with a cultivar at a range of six densities (1.15–25 plants m^−2^); at the highest density the cultivar significantly exceeded the landrace for grain yield (29%) but at the lowest density the reverse was true (32% landrace superiority) [8]. The relationship of yield potential of a genotype with its genetic competitive ability was deliberately investigated in a wheat study [5], where the top at the isolation environment genotypes was top at the crop environment as well but bottom at the competition environment, while the bottom at the isolation environment performed inversely. The results from two intercropping studies are also exceptionally informative. In the sole crop system, the top out of the 10 bean (*Phaseolus vulgaris*) cultivars over-yielded by 400% the bottom one; inversely, in the intercropping system with a maize (*Zea mays*) hybrid the latter was the top and by 27% superior over the former [14]. In another study [15], among four pea (*Pisum sativum*) cultivars as sole crops the best cv. Allround outyielded 150% the worst cv. Salome; however, cv. Salome accumulated significantly greater amounts of N in intercrops with barley (*Hordeum vulgare*) than the other pea cultivars and yielded 53% higher compared to cv. Allround.

Because of the negative association between yield and competitive ability, in the competition environment the value of a landrace as a potential gene pool on which to apply breeding may be severely underestimated. It was found that the actual value of a landrace becomes apparent only when the interplant distance is high enough to ensure absence of any plant-to-plant interference for resource utilization, so that to eliminate the confounding effects of the competitive ability and allow exceptional genotypes to be revealed [8]. Landraces 5 and 6 in the present study are strong supporters of this assertion, thanks to their superiority over the check at the ULD. In terms of the selectable plants (Figure 2(b)), the landrace 6 in particular, a priori eliminable on the ground of its performance in dense stand as poorest performing, gave almost fourfold more selectable plants. Landrace 5 likewise 3 and 4 gave double number of selectable plants compared to the check. Furthermore, the fact that half of the selectable plants exceeded the highest check relative yield shows that landraces include individual genotypes potential to evolve to high yielding pure line cultivars. Moreover, genetic gain in wheat for yield and yield components has been associated more with short- rather than tall-stemmed genotypes; thus if the latter predominate within densely grown populations recognition of the desirable short genotypes becomes uncertain [16]. Consequently, single-plant selection at the ULD appears unique to further utilize the within a landrace existing variability [18].

Breeding at ultralow density is beneficial for numerous reasons [10]. For example, aside from erasing the confounding effects of intergenotypic competition, ultralow density maximizes phenotypic expression and differentiation to facilitate selection. Under very low density, two studies in wheat managed to exploit and turn to advantage even the limited intracultivar genetic variation [5, 17]. It does not exclude good performance at high density on the presupposition that the final outcome is a pure line variety where just intra- and not intergenotypic competition prevails [10, 18]. Indicatively, highly significant correlations (*P* < 0.01) between space-planted nurseries and densely seeded situations were found for a number of traits including yield [19]. Further, it might implement yield compensation components targeting low and stable interseasonally optimum population, an imperative need to bridge current gap between potential and attainable yield and promote crop sustainability and food security [18]. Evidential of the value of breeding in the absence of competition are relevant studies on other crops as well [20–22]. Specifically, single plant selection under very low density within two dry bean landraces proved to be successful in obtaining sister lines that at the farming density and across a range of varying conditions performed stable and yielding significantly higher than their ancestors [20]. The procedure within a lentil (*Lens culinaris*) landrace was successful in development of 2nd generation sister lines of improved health status and potentially virus-tolerant varieties [21].

Concluding, it is demonstrated that a landrace, as a potential germplasm to apply breeding, may be severely underestimated under competition conditions. Different seasons of testing the input regimes might have produced a bias; however, the magnitude of differences particularly for landraces 5 and 6 (Figure 2(a)) allows to infer that the actual value of a landrace becomes apparent only when plant density is low enough to eliminate any plant-to-plant interference for resource utilization and erase the confounding effects of the competitive ability. Competition implies unequal resource use where the strong competitor grows at the expense of the high yielder. However, yield gain from the former fail to compensate for the yield loss of the latter, and the outcome is low yield in overall. This statement comes in agreement with two previous studies [7, 13] which found it necessary to reduce intrapopulation inequality. Competition also justifies the poor performance of a landrace at the TFD; however, the landrace may comprise weak competitors potential to produce highly at pure stands. The Donald “crop” ideotype is genetically homogeneous weak competitor genotype that performs well in monoculture, but does less well when surrounded by plants of the form of the “competition” ideotype [23]. The negative relationship between yield and competitive ability also justifies possible absence of relation between spaced and densely grown plants at early segregating progenies, which may erringly lead to the conclusion that there is no relationship between spaced and densely grown plants [18]. Lastly, the absence of competition appears to be an imperative condition in order to reveal the exceptional genotypes amongst a mixture of genotypes.

## Figures and Tables

**Table 1:** Wheat (*Triticum* spp.) landraces and the check cultivar used.

Code	Name	Species	Date of acquisition	Origination
1	Ntopia Heracleiou-184	*T.durum *	1982	Heraklion/Crete
2	Atsiki-4	*T. durum *	1982	Lemnos
3	Mavragani Samou	*T. durum *	1983	Samos
4	Nteves-35	*T. durum *	1982	Northwest Greece
5	Zoulitsa Arkadias	*T. aestivum *	1982	Arkadia
6	Grinias Zakynthou	*T. aestivum *	1983	Zakynthos
check	cv. Simeto	*T. durum *		GAIA SEEDS S.A
